# Cholesteryl Phenolipids as Potential Biomembrane Antioxidants

**DOI:** 10.3390/molecules29204959

**Published:** 2024-10-20

**Authors:** Vânia Costa, Marlene Costa, Francisca Arques, Mariana Ferreira, Paula Gameiro, Dulce Geraldo, Luís S. Monteiro, Fátima Paiva-Martins

**Affiliations:** 1 REQUIMTE-LAQV, Department of Chemistry and Biochemistry, Faculty of Sciences, University of Porto, 4169-007 Porto, Portugal; vaniasilvacosta@gmail.com (V.C.); marlene.costa@fc.up.pt (M.C.); up202007509@edu.fc.up.pt (F.A.); mariana.ferreira@fc.up.pt (M.F.); agsantos@fc.up.pt (P.G.); 2 Chemistry Centre, University of Minho, Gualtar, 4710-057 Braga, Portugal; gdulce@quimica.uminho.pt (D.G.); monteiro@quimica.uminho.pt (L.S.M.)

**Keywords:** phenolic acids, phenolipids, cholesteryl esters, liposomes, membrane, caffeic acid, protocatechuic acid, homoprotocatechuic acid, dihydrocaffeic acid

## Abstract

The lipophilization of polyphenols (phenolipids) may increase their affinity for membranes, leading to better antioxidant protection. Cholesteryl esters of caffeic, dihydrocaffeic, homoprotocatechuic and protocatechuic acids were synthetized in a one-step procedure with good to excellent yields of ~50–95%. After evaluation of their radical scavenging capacity by the DPPH method and establishing the anodic peak potential by cyclic voltammetry, their antioxidant capacity against AAPH-induced oxidative stress in soybean PC liposomes was determined. Their interaction with the liposomal membrane was studied with the aid of three fluorescence probes located at different depths in the membrane. The cholesteryl esters showed a better or similar radical scavenging capacity to that of α-tocopherol and a lower anodic peak potential than the corresponding parental phenolic acids. Cholesteryl esters were able to protect liposomes to a similar or greater extent than α-tocopherol. However, despite their antiradical capacity and being able to penetrate and orientate in the membrane in a parallel position to phospholipids, the antioxidant efficiency of cholesteryl esters was deeply dependent on the phenolipid polyphenolic moiety structure. When incorporated during liposome preparation, cholesteryl protocatechuate and caffeate showed more than double the activity of α-tocopherol. Thus, cholesteryl phenolipids may protect biomembranes against oxidative stress to a greater extent than α-tocopherol.

## 1. Introduction

Increase in life expectancy has led to an aging population. Current trends indicate that by 2030, people aged 65 and older will represent more than one-fifth of the European Union’s population (The United Nations Economic Commission for Europe). In parallel, the incidence of frailty syndrome in the elderly [1], a geriatric syndrome characterized by reduced resistance, decreased physiological function, and of non-transmissive diseases, has been rising, with high impact on public health and in health costs due to their dramatic consequences: increased risk of multimorbidity, disability, hospitalization, institutionalization and mortality [2]. Metabolic disorders are at the basis of potentially fatal diseases, such as cardiovascular diseases (CVD), diabetes, steatosis and neurodegenerative diseases, and despite the numerous therapies developed, they continue to affect the entire population [3,4,5,6,7]. The causes that lead to these disorders are various and complexly interconnected, as they involve different molecular pathways and gene expression. However, scientific evidence points to two common denominators, oxidative stress and inflammation [8,9,10,11]. Risk factors such as hyperglycemia, increased glycated hemoglobin and triacylglycerols and decreased high-density lipoprotein cholesterol (HDL-C) levels determine the activation of the pro-oxidative NADPH oxidase and mitochondrial disfunction, leading to a high production of oxygen reactive species (ROS). In contrast, there is an inhibition of the antioxidant defense systems, namely a decrease in the activity of superoxide dismutase (SOD), catalase (CAT), and enzymes involved in glutathione (GSH) metabolism, leading to an increase in oxidative stress [8,10]. This oxidative stress subsequently acts as a trigger for the activation of numerous inflammatory factors, namely tumor necrosis factor alpha (TNFα), inflammasome protein 3 (NLRP3), C-reactive protein and nuclear factor kappa B (NF-kB) [8]. Moreover, under the conditions of oxidative stress, the likelihood of oxidation of low-density lipoproteins (LDLs) and HDLs, endothelial cell dysfunction and the formation of crystalline cholesterol aggregates inside membrane bilayers that precipitate the formation of atheroma is increased [11]. Since aging and metabolic diseases are directly related to oxidative stress [1,2], therapeutic interventions using membrane-direct antioxidants may reduce oxidative injuries in cell and organelle membranes and lipoproteins. Furthermore, the development of more efficient and non-toxic antioxidants is also of the greatest importance for the food and cosmetic industry.

Phenolic acids have been pointed out as potential in vivo antioxidants, contributing to the maintenance of the structural integrity of biomembranes, thus reducing the effects of oxidative stress [4]. They have been shown to exhibit robust in vitro pharmacological effects but their low bioavailability, due to their hydrophilic character, limits their use. There bioavailability can be altered by increasing their liposolubility, by graffitiing an alkyl chain by esterification, and these new molecules have been called phenolipids. Phenolipids would, therefore, have an improved ability to interact and cross membranes, leading to greater pharmacological efficacy [5]. Moreover, lipophilization would also allow the use of these antioxidants in lipidic-based formulations such as micellar, emulsified and liposomal systems, which is of particular interest for the food and cosmetic industries. However, this increase in liposolubility also brings to phenolipids challenges to the bioavailability in aqueous media and possible toxicity in vivo. Encapsulation of these compounds inside liposomes or as phytosomes may overcome some of the bioavailability limitations but the possible toxicity will hinder their application.

One of the largest obstacles to the use of antioxidants in the therapy and prevention of diseases caused by oxidative stress is the lack of knowledge about their metabolism and mechanisms of action and the toxicity that can occur in vivo. In theory, and also in practice, all antioxidants, including the natural antioxidant α-tocopherol, can be harmful for living bodies if not used under proper conditions and concentrations [12,13,14,15,16,17]. It is, therefore, of primary importance to understand their protection mechanisms and how these compounds interact with cell constituents.

Although most of the protective mechanisms of natural phenols and phenolipids in vivo have been related to the modulation of several metabolic pathways and gene expressions, direct interactions with biomembranes also need to be considered. Sherratt et al. [18] found that some rosemary acid phenolipids were capable of inhibiting both lipid oxidation, the formation of cholesterol clusters and their crystallization within model membranes. More recently, Lopes et al. also observed a high antioxidant protection from some hydroxytyrosol and caffeic acid phenolipids in model membranes [19,20].

Studies concerning biomembranes and lipoproteins are scarce. Nevertheless, some gallic, caffeic and ferulic acid phenolipids, depending on their alkyl chain length, were shown to protect LDL from oxidative injury [21,22]. Some evaluations have also been conducted using red blood cells (RBCs). These cells are interesting cell models for the evaluation of radical scavenging antioxidants because, on the one hand, RBCs are quite prone to oxidation due to their biological function and, on the other hand, due to the impossibility of gene expression modulation mechanisms occurring. Some authors have observed strong protection activity against AAPH-induced oxidative injury resulting from some phenolipids of hydroxytyrosol and caffeic acid [19,20]. However, this protective activity was not only dependent on the phenolipid alkyl chain length but also on their concentration. Lopez et al. reported an impressive protective capacity against hemolysis from octyl and palmitoyl caffeates (CA-C8 and CA-C16, respectively) at the concentration of only 2.5 μM [20]. However, with the increase in the concentration, the caffeates with lower alkyl chain length increased their protection but the same was not observed with the more lipophilic derivatives CA-C8 and CA-C16. Further investigation found that hemolysis was not caused by oxidation but that these caffeates with long alkyl chains are able to increase the anisotropy of membranes. The decrease in the fluidity of the membrane due to these phenolipids used at higher concentrations would probably lead to a less flexible membrane, more prone to hemolysis [20].

In 2018, and after decades of use, European Regulation 2018/148 banned the use of the more lipophilic gallates, octyl and dodecyl gallates, in foods due to the low number of toxicological studies. In fact, just a few studies are found in the literature concerning the toxicity of phenolipids. In vitro mitochondrial lipid peroxidation induced by Fe(III)-adenosine 5′-diphosphate/reduced nicotinamide adenine dinucleotide was shown to be inhibited by dodecyl gallate, and this phenolipid has protected mitochondrial functions and human red blood cells against oxidative stresses [23]. In contrast, other in vitro toxicity studies have shown that more lipophilic phenolipids such as octyl caffeate, octyl sinapate or octyl and cetyl protocatechuate are highly toxic, even at a very low concentration [16,17]. An impressive result was observed for the toxicity of protocatechuates at the nanomolar range using the macrophage cell line RAW 264.7, a widely used cell line in anti-inflammatory drug screening. Mechanistic studies show that these protocatechuates were able to disrupt mitochondrial membrane potential (ΔΨm), to increase reactive oxygen species (ROS) and to activate the programmed cell death process [17]. Although this toxicity may not be observed in vivo, this preliminary toxicity assessment may result in a low potential for the future use of phenolipids in food and pharmaceutical industries.

**Figure 1:**
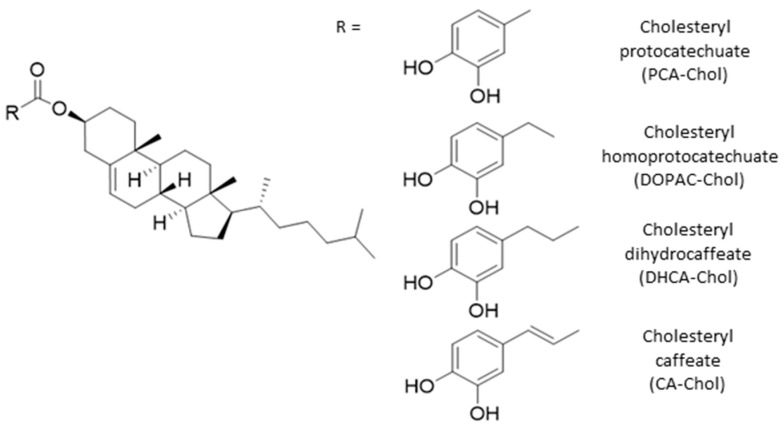
Cholesterol-derived phenolipids.

Recently, there has been a growing interest in natural steryl phenolipids such as steryl ferulates and caffeates found in cereals, as these compounds have shown strong antioxidant activity in edible oils [24] and anti-inflammatory properties [16]. Limited toxicological screening has shown that this class of phenolipids were not toxic to the Caco2 cell lines in contrast with the corresponding octyl and cetyl phenolipids. Therefore, in this work, esters of cholesterol, the most important sterol in animal biomembranes and lipoproteins, with several polyphenolic acids were synthetized (Figure 1). This may constitute a promising strategy to fine-tune high free radical scavenging molecules at biomembranes and, with this, provide a higher antioxidant protection of biomembranes without deeply affecting the membrane dynamics, thus with a potential lower toxic activity.

## 2. Results and Discussion

The previously reported methods for the synthesis of phenolic acid phenolipids usually involves, at least, an initial protection step of the phenolic hydroxyl groups, a transesterification or an esterification step using coupling agents in basic media, and a final deprotection step to afford the final wanted ester [25,26]. The need for hydroxyl group protection is essential when working in basic conditions, not only to prevent the preferential esterification of these groups but, also in the case of polyphenols, to also prevent their easy oxidation. The esterification step of polyphenolic acids involving secondary alcohols is also challenging since these alcohols are less reactive due to being more sterically hindered. Moreover, previous tentative esterifications of polyphenolic acids, such as caffeic acid, using enzymatic catalysis have failed [27]. Usually, lipases have low efficiency when the substrate is a secondary alcohol, and although it was possible to set conditions for the synthesis of several steryl ferulates in high yields, the same conditions were only able to afford steryl caffeates in very low yields (~16%) [28]. Therefore, in this work we developed new synthetic procedures for the synthesis of cholesteryl caffeate, dihydrocaffeate, homoprotocatechuate and protocatechuate, which provide high yields in just one step, by the direct esterification of phenolic acids with cholesterol under acidic conditions.

The reaction solvent proved to be crucial for the success of the esterification. On one hand, a less polar solvent favors the esterification reaction but on the other hand, the low solubility of phenolic acids in less polar solvents prevents the compounds from reacting. Therefore, phenolic acids and cholesterol in toluene did not react under acidic catalysis, probably due to the low solubility of the phenolic acids in this solvent, with the exception of PCA that reacted with a modest yield (~20%). In contrast, using THF as solvent, all reagents were soluble, and the cholesteryl esters were obtained in good yields (50–70%), except for the PCA that did not react either in this solvent or in acetonitrile under the same conditions. Therefore, another strategy was used for PCA ester synthesis, and this less reactive acid was made to react with cholesterol as a chloride. Thus, protocatechuate ester synthesis was achieved by first reacting PCA with thionyl chloride in dichloromethane followed by the addition of cholesterol, in a one-pot synthesis.

Although cholesteryl caffeate could be synthesized by a similar procedure to that used in the synthesis of cholesteryl dihydrocaffeate and with similar yields, in this case a two-step synthetic route was preferred. By using a cholesteryl monomalonate and 3,4-dihydroxybenzaldehyde through a Verley–Doebner modification of the Knoevenagel condensation [29,30], cholesteryl caffeate could be obtained in global yields of over 95%.

In all these procedures, the reaction time was relatively long (5 days), but this inconvenience was counterbalanced by the smaller number of synthetic and easier purification steps.

### 2.1. Evaluation of the Potential Free Radical Scavenging Capacity of Cholesteryl Esters

Before determining the cholesteryl esters’ protective efficiency in a membrane model, we investigated the effect of the cholesteryl moiety on the radical scavenging capacity of the cholesteryl esters against the DPPH radical by determining their corresponding EC_50_ value, and on the redox potentials (*Ep*) in bulk solution (Table 1 and Table 2) [20].

The radical scavenging capacity against the DPPH radical decreased with the introduction of the cholesteryl moiety when compared with the parental phenolic acid with the exception of the PCA ester that kept the same or increased capacity, depending on the time of reaction. The introduction of a bulky group, such the cholesteryl moiety, probably causes a steric hinderance for the reaction of the aromatic hydroxyl group with the also bulky radical DPPH. However, in the case of the cholesteryl protocatechuate, the decrease in the reactivity due to steric hinderance is maybe compensated by the increase in the reactivity of the aromatic hydroxyl group caused by the esterification of the carboxylic group directly linked to the ring. Nevertheless, and besides this general decrease in the radical scavenging capacity, cholesteryl esters showed better or similar radical scavenging capacity when compared with α-tocopherol.

**Figure 2:**
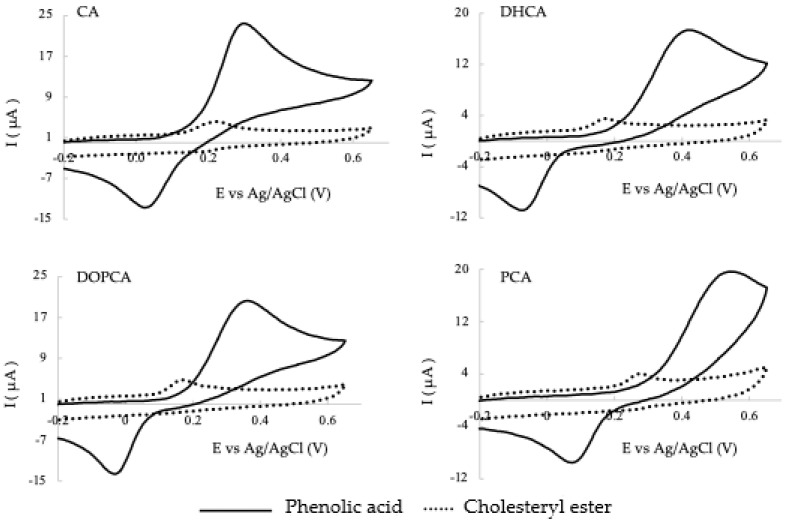
Cyclic voltammograms obtained in a phosphate buffer (pH 7.3) using a glassy carbon electrode at 100 mV/s for 0.10 mM of phenolic acids (CA, DHCA, DOPCA, PCA) and their corresponding cholesteryl esters.

Antioxidants exert their activity mainly through electron transfer mechanisms, with oxidation potential being a key indicator of their efficacy. To rank the antioxidant capacities of the novel compounds, cyclic voltammetry was also employed as a characterization technique as the oxidation peak potential obtained provides insight into the oxidation susceptibility of each antioxidant. Figure 2 shows the overlay of the voltammograms for the phenolic acids and their corresponding cholesteryl esters.

All phenolic acids exhibit low oxidation potentials (Figure 2, Table 2), with a cathodic peak observed in the return sweep showing that the process follows a reversible mechanism. Among the compounds studied, CA exhibits the highest antioxidant power, indicated by the lowest value of oxidation peak potential. In contrast, PCA shows the highest oxidation peak potential, and the greatest separation between the anodic and cathodic peaks, showing in this case a more difficult electronic transfer and, therefore, a less reversible mechanism. Esterification of the antioxidants with cholesterol significantly lowers the anodic peak potential for all four compounds. This variation in anodic potential is consistent for all compounds, except for CA, which shows a considerably smaller change. However, CA already had the lowest oxidation potential (Table 2) and so the effect of introducing the cholesterol moiety is smaller. The lower the anodic potential value, the easier it is to supply the electron to a free radical. Therefore, in contrast with the results obtained by the radical scavenging capacity against the DPPH radical, the esterification significantly increases the potential capacity of compounds to scavenge free radicals, with DHCA and DOPAC esters having the highest and the PCA ester having the lowest capacity. Additionally, esterification results in a smaller intensity of the anodic peak due to the increase in the volume of the compound that decreases their diffusion coefficient. Moreover, no cathodic peak was detected for all the cholesteryl derivatives, showing that the processes are not reversible in the case of the esters.

### 2.2. Interaction of Compounds with Liposomal Membranes

Studies concerning the cell uptake of compounds have shown that polyphenol liposolubility plays an important role in their capacity to cross membranes and, therefore, in their bioactivity. Thus, there has been a high interest in the search for new lipophilic derivatives of simple polyphenols, known as phenolipids. In this work, the choice of cholesterol as the lipophilic moiety of the synthetized phenolipids was made, taking into account that this sterol is the most important sterol found in animal cells and that it is found often in equimolar quantities in regard to phospholipids, playing an important role in membrane microfluidity. Thus, at low temperatures, cholesterol reduces the membrane high solid order phase, while at higher temperatures, it reduces the high liquid-disorder phase [31]. Therefore, this molecule found in all animal biomembranes and lipoproteins acts as a buffer, preventing oscillations in their microfluidity caused by temperature variations. Its particular structure and location in cell membranes contributes to this property, with its hydroxyl group next to the polar heads of the phospholipids, the polycyclic structure located in a more intermediate zone of the monolayer and thus reducing the fluidity in this region, and a side chain (the 6 -methylheptan-2-yl group at the C17) which provides fluidity to the innermost zone of the monolayer [31].

The increase in the liposolubility of the phenolic acids, conferred by the introduction of the cholesterol moiety (evaluated by the miLog *P*), leads to values similar to that of α-tocopherol (Table 1) and is expected to increase the interaction of these compounds with the lipid bilayer. Thus, in order to understand how these compounds interact with membrane lipids, two simplified models of lipidic membranes, L-α–dimyristoylphosphatidylcholine (DMPC) and soy L-α-phosphatidylcholine (PC) liposomes [32,33], and three different membrane fluorescent probes, 2-(9-anthroyloxy)stearic acid (2-AS), 1,6-diphenyl-1,3,5-hexatriene-4′-trimethylammonium tosylate (TMA-DPH) and 1,6 diphenyl 1,3,5 hexatriene (DPH), which are located at different depths in the liposome membrane, were used. Data obtained by parallax analysis revealed that these probes are located at 16, 10.9 and 7.8 Å, respectively, from the center of the bilayer [34].

**Figure 3:**
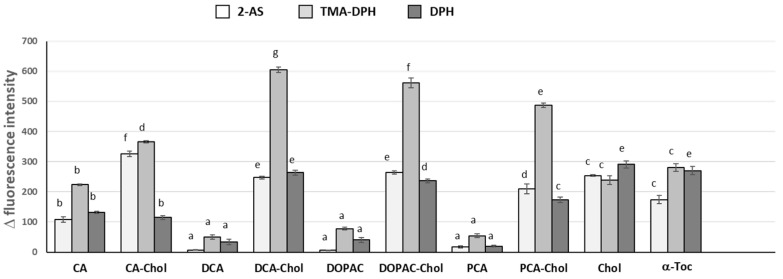
Quenching effect of antioxidants on 2-AS, TMA-DPH and DPH emission intensity. Different letters for each probe represent statistically different means, *p* < 0.05.

Fluorescence quenching studies (Figure 3) using liposomes containing the 2-AS, TMA-DPH or the DPH probes show the quenching of the fluorescence emission of the probes in the presence of the compounds.

**Figure 4:**
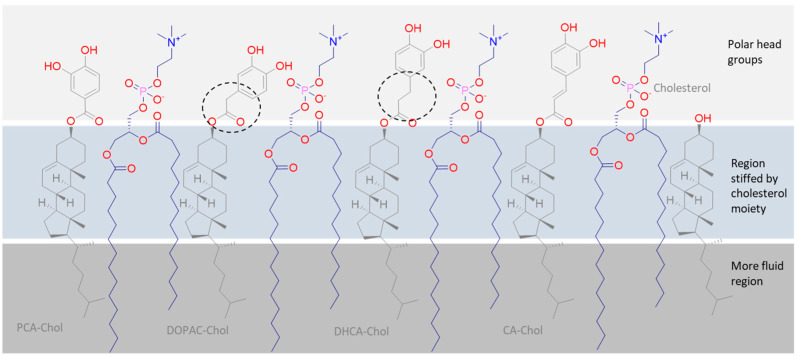
Representative arrangement of cholesteryl esters embedded in the phosphatidylcholine membrane monolayer.

In general, parental phenolic acids showed a very small quenching effect over the three probes, except caffeic acid, proving their more external location and low interaction with the membrane. However, caffeic acid was able to have an important interaction with the 3 probes, showing that this phenolic acid has a limited but higher capacity of penetrating membranes, probably due to its lower water solubility when compared with the other phenolic acids. In contrast, a sharp decrease in the three probes’ fluorescence intensity over a period of a few seconds was observed after adding the cholesteryl esters, cholesterol and α-tocopherol to the DMPC liposomes suspensions, followed by a stabilization in the value. These observations show that there was a very fast incorporation of all cholesteryl esters in the liposomal membrane and, since these compounds were able to interact along the entire length of the membrane lipid monolayer, these compounds must orientate in the membrane in a parallel position to phospholipids (Figure 4).

Nevertheless, the interactions observed for the different phenolipids with the three probes did not show the same intensity. CA-Chol showed the lowest interaction with the DPH probe but the highest with the 2-AS probe, due probably to a less deep position in the monolayer. On the other hand, DCA-Chol, DOPAC-Chol and PCA-Chol showed higher interaction with the TMA-DPH and with the DPH probe, due probably to a deeper position. Both cholesterol and α-tocopherol showed interactions with the three probes but much less interaction with the TMA-DPH probe, located below the polar head of the phospholipids.

**Figure 5:**
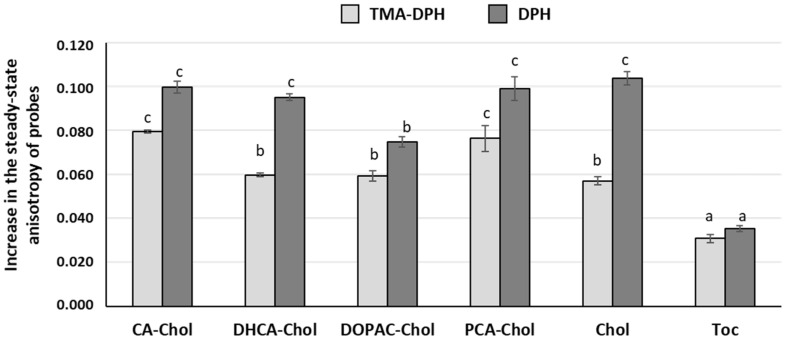
Changes in the steady-state fluorescence anisotropy of probes TMA-DPH and DPH incorporated in DMPC LUVs in the presence of compounds (200 μM). Different letters for each probe represent statistically different means, *p* < 0.05.

As membrane fluidity is very important for biomembrane function, the fluidity of the DMPC LUVs model membrane in the presence of cholesteryl esters, CA, cholesterol and α-tocopherol, were evaluated at 37 ± 0.1 °C. The data are reported as changes in the steady-state fluorescence anisotropy of the TMA-DPH and DPH probes [19] (Figure 5).

The data obtained demonstrated that, at the concentration tested, cholesteryl esters increased the anisotropy values of the probes located either in the more external and deeper regions of the monolayer, meaning that all compounds were able to decrease the fluidity in these areas of membrane. However, this decrease was similar to that obtained for cholesterol at the same concentration. Although α-tocopherol also increased the anisotropy value of both probes, this increase was smaller when compared to cholesterol and its esters. Once more, these results indicate that cholesteryl esters must be aligned relative to the membrane normal. In contrast, and despite the small interaction with the three probes observed for caffeic acid, this compound was not able to significantly change the steady-state anisotropy parameter for the TMA-DPH and DPH probes [20].

### 2.3. Antioxidant Capacity of Cholesteryl Esters in Liposomal Systems

**Figure 6:**
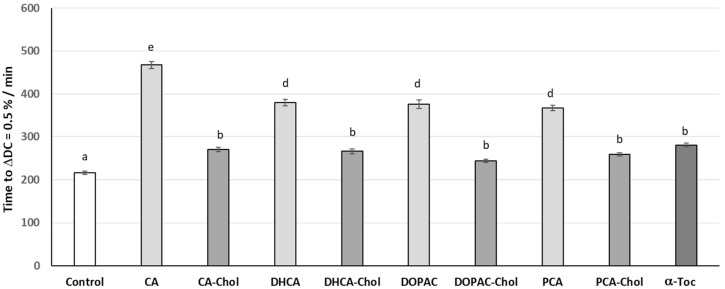
Time in minutes for liposomes incubated with antioxidants at 0.75 μM to reach the conjugated diene content of 0.5%. Error bars represent standard deviation. Different letters represent statistically different means, *p* < 0.05.

Hydroperoxyl radicals (HOO^•^), from the aqueous phase, are important species for initiating lipid and protein oxidation in the membranes [35]. Therefore, the use of the 2,2′-azo-bis(2-amidinopropane) dihydrochloride (AAPH) as a water-soluble free radical initiator has been widely used for biological studies. By reacting with oxygen, this azo compound produces peroxyl radicals at a constant rate that recreates the attack of free radicals such as hydroperoxyl radicals and other possible peroxyl radicals on biomembranes [35]. When the antioxidant compounds (AOs) were incubated with preformed liposomes in the presence of AAPH, the oxidative stability of liposomal suspensions increased (Figure 6) when compared with the control. However, the protection given by the compounds was not the same, and the antioxidant capacity of the parental phenolic acids was better than that of the cholesteryl esters or α-tocopherol. The order of antioxidant activity was CA > DHCA~DOPAC~PCA > α-Toc~CA-Chol~PCA-Col~DOPAC-Chol > control (Figure 6).

One of the reasons for the lower antioxidant activity of the cholesteryl esters and α-tocopherol is probably their very low hydrosolubility, confirmed by the very high miLog *p* values (Table 1). In fact, these AOs were shown to be soluble in ethanol but not in methanol or PBS. Therefore, compounds were dissolved in dimethyl sulfoxide (DMSO), taking into account that, when added to the liposome suspension, the final percentage of DMSO in the liposome suspension was lower than 2%. During this procedure, however, aggregation of these lipophilic compounds is likely to occur when the molecules enter in contact with the PBS solution. This may hinder the penetration of these molecules into the liposome membrane and, therefore, the actual concentration of compounds in the membrane may be much lower than the stochiometric concentration and, consequently, their antioxidant efficiency is lower than expected. Another explanation can be related to the fact that a hydrosoluble free radical initiator is being used. For this reason, the scavenging of radicals before the attack at the membrane may be more efficient by the more hydrosoluble compounds. Further studies with a lipophilic radical scavenging initiator may unravel the reason for these results.

**Figure 7:**
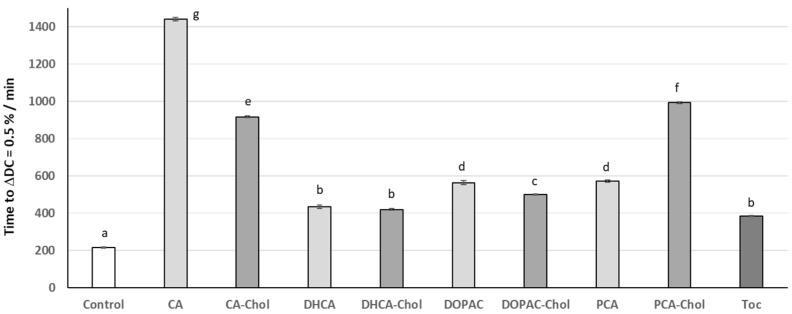
Time in minutes for liposomes with incorporated AOs at 0.75 μM to reach the conjugated diene content of 0.5%. Error bars represent standard deviation. Different letters represent statistically different means, *p* < 0.05.

A further stability assay was performed, now with the incorporation of compounds during liposome preparation (Figure 7).

With this procedure, and since the compounds were previously dissolved together with phospholipids in an organic solvent, the incorporation of compounds in the membrane would not be so affected by the very low hydrosolubility of these compounds in the PBS buffer since strong interactions of compounds with the phospholipid bilayer may be established before the liposome formation. Moreover, the polar heads of phospholipids do not serve as a barrier to the penetration of compounds to the more lipophilic zone of the membrane, and the absence of water molecules makes the establishment of hydrogen and ionic bounds easier between the phenolic acid hydroxyl and carboxylic groups and the polar phospholipid heads. When the AO was incorporated during the preparation of the PC liposome, the oxidative stability against AAPH induced oxidative injury increased for all suspensions (Figure 7), making the order of antioxidant activity now different to the previous stability study: CA >> PCA-Chol > CA-Chol > DOPAC~PCA > DOPAC-Chol > DHCA~DHCA-Chol~α-Toc >> control. In this case, the liposomal suspensions containing PCA-Chol and CA-Chol showed more than double the stability of the suspension containing α-tocopherol. On the other hand, the stability of liposomes containing DHCA-Chol or DOPAC-Chol, although similar or slightly higher than the those containing α-tocopherol, were similar or even lower than that of the corresponding parental phenolic acid. According to these results, the lipophilization of DCHA and DOPAC with the cholesterol moiety did not increase the capacity of compounds in protecting the membrane from oxidative injury. Once these compounds were actually the cholesteryl esters with the highest potential to act as radical scavenging antioxidants, the observed results can only be explained by the worst availability of their aromatic hydroxy groups to act as radical scavengers. According to the structure of these compounds, they only differ from the PCA-Chol and CA-Chol in the side arm of the phenolic acid moiety. Apparently, the more flexible arm (indicated in Figure 4 by a circle) may allow these compounds to bend and establish strong interactions with the phospholipidic polar groups, making the hydroxyl groups less available for radical scavenging. These compounds also showed a higher interaction with the TMA-DPH probe (a cationic probe) when added to the liposomes (Figure 3). This may confirm the possibility of a higher interaction of the phenolic moiety in a more inner zone of the membrane. On the other hand, the more flexible side arm may also lead to a less favorable conformation. The rigidity of the protocatechuic and caffeic acid moieties seems to confer to these compounds a more favorable position in the membrane, leading to better antioxidant capacity.

The result obtained for caffeic acid was surprising. Liposomes prepared with this phenolic acid showed the highest oxidative stability. As already mentioned, the preparation of liposomes in the presence of compounds allows for the establishment of better interactions of these with the phenolipid head groups as the absence of water during the liposomal film preparation does not hinder the interaction between molecules. Apparently, the more planar and less flexible molecule of CA seems to be able to better interact with the polar head groups and shield the membrane in a more efficient way than the other phenolic acids, thus being more capable of scavenging free radicals arriving from the aqueous phase.

## 3. Materials and Methods

### 3.1. Materials

Cholesterol (Chol, 95%), α-tocopherol (α-Toc, 95%), thionyl chloride (99.7%) and 3,4-dihydroxybenzoic acid (PCA, 97%) were obtained from Thermo Fisher Scientific. 3,4-dihydroxycinnamic acid (CA), 3,4-dihydroxybenzaldeyde, β-Alanine (99%), 2,2-diphenyl-1-picrylhydrazyl (DPPH•, 95%) and 1,6-diphenyl-1,3,5-hexatriene (DPH) were purchased from Sigma-Aldrich. 3,4-dihydroxyphenylacetic acid (DOPAC, >98%) and Meldrum’s acid (>98%) were acquired from TCI. *p*-Toluene-sulfonic acid (99%) and pyridine (99.5%) were bought from Merck. Sodium sulfate anhydrous (99.9%) and lecithin from soybean (PC Soy 90%) were purchased from Panreac AppliChem. 1,6-diphenyl-1,3,5-hexatriene-4′-trimethylammonium tosylate (TMA-DPH) and hydrochloric acid (37%) were obtained from Fluka. 3-(3,4-dihydroxyphenyl)propionic acid (DHCA, >98%), 2-(9-anthroyloxy)stearic acid (2-AS) and 1,2-dimyristoyl-sn-glycero-3-phosphocholine (DMPC) were from Alfa Aesar, Molecular Probes and Avanti Polar Lipids, respectively. All organic solvents used were analytical or HPLC grade and were obtained from Fisher Scientific.

### 3.2. Synthesis of Cholesteryl Esters of Polyphenolic Acids

All reactions and purifications were followed by thin layer chromatography (TLC), using silica gel plates on fluoroChem 60 F254 aluminum support with development in an iodine chamber. The retention factor (Rf) of the synthetized compounds was determined. Syntheses were also followed by high-performance liquid chromatography (HPLC), using Vanquish equipment from Thermoscientific, with a Diode-array detector and the Chromeleon 7 software. A LiChrospher^®^ 100 RP-18 (5 μm) LiChroCART^®^ 250-4 reverse-phase column (Merck, Lisbon, Portugal) and a LiChrospher^®^ 100 RP-18 (5 μm) LiChroCART^®^ 4-4 precolumn were used. Column and sample compartment temperatures were set to 30 °C. The samples (10 μL) were analyzed for 30 min, using 50% ACN and 50% MeOH as eluents at a flow rate of 1.0 mL/min, with UV detection at 280 nm. Prior to analysis, the samples were filtered using polyvinylidene fluoride (PVDF) syringe filters with a porosity of 0.22 μm (Branchia). HPLC analyses were used to determine the retention time (RT) and the maximum absorption wavelength (λ_max_) of the compound.

The final compounds were identified and characterized by Proton and Carbon Nuclear Magnetic Resonance (^1^H-NMR and ^13^C-NMR, respectively), using deuterated chloroform or acetone (VWR) as solvent. NMR analyses were performed by the Centre for Materials of the University of Porto (CEMUP), using a Bruker Advance III NMR spectrometer. Chemical shifts (δ) were reported as parts per million (ppm), and coupling constants (J) were given in hertz (Hz).

Higher Resolution Mass Spectroscopy (HRMS) was used to confirm the exact mass of the cholesteryl esters. Analysis was conducted on an Orbitrap Exploris 120 mass spectrometer (Thermo Fischer Scientific, Bremen, Germany) controlled by Orbitrap Exploris Tune Application 2.0.185.35 and Xcalibur 4.4.16.14. The resolution of the SIM MS scan was 60,000.

Melting points were measured using a SMP1 Stuart Scientific melting point apparatus.

#### 3.2.1. Synthesis of Cholesteryl Caffeate

The synthesis of cholesteryl caffeate was achieved by a two-step protocol [29,30]. Firstly, the synthesis of cholesteryl monomalonate was performed using Meldrum’s acid, and then the monomalonate was made to react with 3,4-dihydroxybenzaldehyde by a Knoevenagel condensation in order to achieve the cholesteryl caffeate.

Briefly, equimolar quantities (2.6 mmol) of Meldrum’s acid and cholesterol were refluxed in 6 mL of toluene for 4 h. Then, the solvent was evaporated, and the cholesteryl monomalonate was obtained as a white solid (~99%; Rf = 0.91; UV-Vis (CH_3_OH) λ_max_ 285 nm) and was used in the synthesis of caffeate, without further purification. ^1^H-NMR (400 MHz, CDCl_3_): δ 5.39 (m, 1H, H-6); 4.71 (m, 1H, H-3); 3.41 (s, 2H, H-29); 2.33 (m, 2H, H-4); 1.49 (m, 26H, H-1, H-2, H-7–H-9, H-11, H-12, H-14–H-17, H-20, H-22–H-25); 1.02 (s, 3H, H-19); 0.91 (d, J_21,20_ = 6.5 Hz, 3H, H-21); 0.87 (d, J_26,25_ = 6.6 Hz, 3H, H-26); 0.86 (d, J_27,25_ = 6.6 Hz, 3H, H-27); 0.68 (s, 3H, H-18). ^13^C-NMR (100 MHz, CDCl_3_): δ 168.81 (C-28); 167.94 (C-30); 139.22 (C-5); 123.37 (C-6); 76.45 (C-3), 56.83 (C-14); 56.29 (C-17); 50.15 (C-9); 42.47 (C-13); 40.15 (C-29); 39.86 (C-12); 39.67 (C-24); 37.96 (C-4); 37.00 (C-10); 36.71 (C-1); 36.33 (C-20); 35.94 (C-22); 32.05 (C-8); 31.99 (C-7); 28.37 (C-2); 28.16 (C-25); 27.69 (C-15); 24.43 (C-16); 23.96 (C-23); 22.97 (C-26); 22.71 (C-27); 21.18 (C-11); 19.43 (C-19); 18.87 (C-21); 12.01 (C-18).

Cholesteryl caffeate was obtained by reacting the monomalonate previously obtained with 3,4-dihydroxybenzaldehyde by a Knoevenagel condensation, following a previously reported procedure with minor modifications [29,30]. Equimolar quantities (2.4 mmol) of monomalonate and 3,4-dihydroxybenzaldehyde were reacted in 6.0 mL of pyridine with 29 mg of β-alanine at 50 °C for 5 days. The reaction mixture was cooled in an ice bath, and 15 mL of concentrated HCl was added. Then, 80 mL of deionized water was added to the mixture and the aqueous phase was extracted with diethyl ether. The organic phase was then dried over anhydrous Na_2_SO_4_, and the solvent was evaporated. The final compound was purified using flash column chromatography over silica gel with petroleum ether/diethyl ether (1:4, *v*/*v*) as eluent. The cholesteryl caffeate was obtained as a white solid (98%; mp 211–213 °C); Rf = 0.86; UV-Vis (CH_3_OH) λ_max_ 217, 245, 299, 326 nm). ^1^H-NMR (400 MHz, (CD_3_)_2_CO) (Figure S1): δ 8,29 (s, 2H, -OH); 7.53 (d, J_37,38_ = 15.9 Hz, 1H, H-37); 7.16 (d, J_36,30_ = 2.0 Hz, 1H, H-36); 7.04 (dd, J_30,31_ = 8.2 Hz, J_30,36_ = 2.0 Hz, 1H, H-30); 6.87 (d, J_31,30_ = 8.2 Hz, 1H, H-31); 6.26 (d, J_38,37_ = 15.9 Hz, 1H, H-38); 5.41 (m, 1H, H-6); 4.64 (m, 1H, H-3); 2.38 (m, 2H, H-4); 1.53 (m, 26H, H-1, H-2, H-7–H-9, H-11, H-12, H-14–H-17, H-20, H-22–H-25); 1.80 (s, 3H, H-19); 0.96 (d, J_21,20_ = 6.5 Hz, 3H, H-21); 0.88 (d, J_26,25_ = 6.6 Hz, 3H, H-26); 0.87 (d, J_27,25_ = 6.6 Hz, 3H, H-27); 0.74 (s, 3H, H-18); 13C-NMR (100 MHz, (CD_3_)_2_CO) (Figure S2): δ 166.82 (C-28); 148.73 (C-35); 146.34 (C-38); 145.51 (C-32); 140.85 (C-5); 127.72 (C-29); 123.15 (C-6); 122.48 (C-30); 116.39 (C-31); 116.12 (C-36); 115.22 (C-37); 74.23 (C-3), 57.61 (C-14); 57.06 (C-17); 51.10 (C-9); 43.12 (C-13); 40.65 (C-12); 40.27 (C-24); 39.07 (C-4); 37.92 (C-10); 37.42 (C-1); 36.98 (C-20); 36.63 (C-22); 32.75 (C-8); 32.64 (C-7); 28.94 (C-2); 28.71 (C-25); 28.67 (C-15); 24.95 (C-16); 24.55 (C-23); 23.09 (C-26); 22.85 (C-27); 21.78 (C-11); 19.70 (C-19); 19.16 (C-21); 12.25 (C-18); HRMS (ESI): *m*/*z* calculated for C_36_H_52_O_4_ = 548.3866; found = 548.3791.

#### 3.2.2. Synthesis of Cholesteryl Dihydrocaffeate and Homoprotocatechuate

Cholesteryl dihydrocaffeate and homoprotocatechuate were synthesized through an esterification reaction using acid catalysis, based on a previously published procedure [19]. In detail, 6 mmol of DHCA or DOPAC and 12 mmol of cholesterol were dissolved in 15 mL of THF containing 0.150 g of *p*-toluene-sulfonic acid and stirred for 5 days at 75 °C. Then, the organic solvent was evaporated and the final compound was purified using flash column chromatography over silica gel with petroleum ether/diethyl ether (1:4, *v*/*v*) as eluent.

*Cholesteryl dihydrocaffeate.* The product was obtained as a white solid (60%, mp 158–160 °C); Rf = 0.89; UV-Vis (CH_3_OH) λ_max_ 202, 282 nm; ^1^H-NMR (400 MHz, CDCl_3_) (Figure S3): 6.76 (d, J_36,30_ = 2.0 Hz, 1H, H-36); 6.71 (dd, J_30,31_ = 8.0 Hz, J_30,36_ = 2.0 Hz, 1H, H-30); 6.59 (d, J_31,30_ = 8.0 Hz, 1H, H-31); 6,15 (s, 1H, -OH); 6,04 (s, 1H, -OH); 5.35 (m, 1H, H-6); 4.59 (m, 1H, H-3); 2.81 (t, J_37,38_ = 7.8 Hz, 2H, H-37); 2.53 (t, J_38,37_ = 7.8 Hz, 2H, H-38); 2.27 (m, 2H, H-4); 1.48 (m, 26H, H-1, H-2, H-7–H-9, H-11, H-12, H-14–H-17, H-20, H-22–H-25); 1.00 (s, 3H, H-19); 0.90 (d, J_21,20_ = 6.5 Hz, 3H, H-21); 0.87 (d, J_26,25_ = 6.6 Hz, 3H, H-26); 0.86 (d, J_27,25_ = 6.6 Hz, 3H, H-27); 0.66 (s, 3H, H-18). ^13^C-NMR (100 MHz, CDCl_3_) (Figure S4): δ 172.82 (C-28); 143.98 (C-35); 142.38 (C-32); 139.78 (C-5); 133.37 (C-29); 122.77 (C-6); 120.48 (C-30); 115.40 (C-31); 115.27 (C-36); 74.24 (C-3), 56.82 (C-14); 56.28 (C-17); 50.15 (C-9); 42.45 (C-13); 39.86 (C-12); 39.65 (C-24); 38.22 (C-4); 37.11 (C-10); 36.72 (C-38); 36.65 (C-1); 36.31 (C-20); 35.92 (C-22); 32.03 (C-8); 31.99 (C-7); 30.56 (C-37); 28.35 (C-2); 28.13 (C-25); 27.88 (C-15); 24.41 (C-16); 23.96 (C-23); 22.93 (C-26); 22.68 (C-27); 21.16 (C-11); 19.43 (C-19); 18.84 (C-21); 11.98 (C-18). HRMS (ESI): m/z calculated for C_36_H_54_O_4_ = 550.4022; found = 550.3946.

*Cholesteryl homoprotocatechuate*. The product was obtained as a white solid (70%, mp 166–168 °C); Rf = 0.79; UV-Vis (CH_3_OH) λ_max_ 202, 283 nm; ^1^H-NMR (400 MHz, CDCl_3_) (Figure S5): δ 6.76 (d, J_36,30_ = 2.0 Hz, 1H, H-36); 6.75 (dd, J_30,31_ = 8.0 Hz, J_30,36_ = 2.0 Hz, 1H, H-30); 6.66 (d, J_31,30_ = 8.0 Hz, 1H, H-31); 5,88 (s, 1H, -OH); 5,62 (s, 1H, -OH); 5.35 (m, 1H, H-6); 4.63 (m, 1H, H-3); 3.47 (s, 2H, H-37); 2.32 (m, 2H, H-4); 1.44 (m, 26H, H-1, H-2, H-7–H-9, H-11, H-12, H-14–H-17, H-20, H-22–H-25); 1.01 (s, 3H, H-19); 0.91 (d, J_21,20_ = 6.5 Hz, 3H, H-21); 0.87 (d, J_26,25_ = 6.7 Hz, 3H, H-26); 0.86 (d, J_27,25_ = 6.7 Hz, 3H, H-27); 0.67 (s, 3H, H-18). ^13^C-NMR (100 MHz, CDCl3) (Figure S6): δ 172.45 (C-28); 143.86 (C-35); 143.13 (C-32); 139.63 (C-5); 126.73 (C-29); 122.97 (C-6); 121.85 (C-30); 116.52 (C-31); 115.52 (C-36); 75.03 (C-3), 56.83 (C-14); 56.30 (C-17); 50.15 (C-9); 42.47 (C-13); 41.15 (C-37); 39.87 (C-12); 39.67 (C-24); 38.15 (C-4); 37.08 (C-10); 36.73 (C-1); 36.34 (C-20); 35.94 (C-22); 32.05 (C-8); 32.00 (C-7); 28.37 (C-2); 28.16 (C-25); 27.86 (C-15); 24.43 (C-16); 23.99 (C-23); 22.96 (C-26); 22.71 (C-27); 21.18 (C-11); 19.46 (C-19); 18.87 (C-21); 12.00 (C-18). HRMS (ESI): m/z calculated for C_35_H_52_O_4_ = 536.3866; found = 536.3781.

#### 3.2.3. Synthesis of Cholesteryl Protocatechuate

Cholesteryl protocatechuate was synthesized following a one-pot procedure. PCA was converted to its corresponding acid chloride by reaction with thionyl chloride. The resulting protocatechuate acid chloride was then added to cholesterol to form the respective ester. Briefly, a solution of PCA (6.5 mmol) in 20 mL of dichloromethane containing 13 mmol of pyridine was stirred and cooled in an ice bath. Then, 13 mmol of thionyl chloride was added dropwise to the mixture. After 24 h of reaction, 13 mmol of cholesterol was added and the mixture was kept at 4 °C for 5 days. Then, the solvent was evaporated, and the final compound was purified using flash column chromatography over silica gel with petroleum ether/diethyl ether (1:4, *v*/*v*) as eluent.

The product was obtained as a white solid (50%, mp 209–211 °C); Rf = 0.89; UV-Vis (CH_3_OH) λ_max_ 205, 260, 295 nm; ^1^H-NMR (400 MHz, CDCl_3_) (Figure S7): δ 7.61 (d, J_36,30_ = 2.0 Hz, 1H, H-36); 7.56 (dd, J_30,31_ = 8.3 Hz, J_30,36_ = 2.0 Hz, 1H, H-30); 6.89 (d, J_31,30_ = 8.3 Hz, 1H, H-31); 6,37 (s, 1H, -OH); 6,24 (s, 1H, -OH); 5.40 (m, 1H, H-6); 4.80 (m, 1H, H-3); 2.44 (m, 2H, H-4); 1.45 (m, 26H, H-1, H-2, H-7–H-9, H-11, H-12, H-14–H-17, H-20, H-22–H-25); 1.06 (s, 3H, H-19); 0.92 (d, J_21,20_ = 6.6 Hz, 3H, H-21); 0.87 (d, J_26,25_ = 6.6 Hz, 3H, H-26); 0.86 (d, J_27,25_ = 6.6 Hz, 3H, H-27); 0.69 (s, 3H, H-18); ^13^C-NMR (100 MHz, CDCl3) (Figure S8): δ 166.22 (C-28); 148.76 (C-35); 143.24 (C-32); 139.84 (C-5); 123.79 (C-29); 123.41 (C-6); 122.92 (C-30); 116.71 (C-31); 114.86 (C-36); 74.76 (C-3), 56.86 (C-14); 56.32 (C-17); 50.21 (C-9); 42.49 (C-13); 39.91 (C-12); 39.68 (C-24); 38.40 (C-4); 37.19 (C-10); 36.81 (C-1); 36.35 (C-20); 35.96 (C-22); 32.09 (C-8); 32.05 (C-7); 28.39 (C-2); 28.17 (C-25); 28.06 (C-15); 24.45 (C-16); 24.00 (C-23); 22.97 (C-26); 22.71 (C-27); 21.21 (C-11); 19.52 (C-19); 18.88 (C-21); 12.02 (C-18). HRMS (ESI): *m*/*z* calculated for C_34_H_50_O_4_ = 522.3709; found = 522.3717.

### 3.3. Determination of milogP Values

The lipophilicity of the compounds was estimated by the miLogP values, calculated using the Molinspiration property engine v2022.08 (Molinspiration Cheminformatics, Slovensky Grob, Slovakia, accessed on 7 July 2024).

### 3.4. DPPH Radical Scavenging Capacity

The DPPH• assay was used to evaluate the radical scavenging capacity of the compounds, as described previously with some modifications [30]. Briefly, 50 μL of AO stock solution in EtOH with increasing final concentrations (3–25 μM) was added to each well in a 96-well microplate. The reaction was started by adding 250 μL of DPPH (final concentration, 112 μM) in each well and the absorbance was recorded at 5 min intervals for 60 min at 25 °C. The relative free antiradical activity was given by the EC_50_ value, defined as concentration of AO required to lower the initial DPPH• concentration by 50%. Absorbance values at four different time intervals (5, 15, 30 and 60 min) were used to calculate EC_50_. Each compound was tested in duplicate in five independent experiments.

### 3.5. Cyclic Voltammetry

Voltametric experiments were carried out with an Autolab PGSTAT 30 potentiostat (Eco-Chemie) with the GPES 4.9 software package (General Purpose Electrochemical Experiments). A three-electrode cell was employed, consisting of a glassy carbon electrode (GCE) as the working electrode, a platinum wire as the counter electrode, and an Ag/AgCl (KCl 1M) electrode as the reference. The scan rate was set at 100 mV/s.

To prepare solutions for determining the peak potentials of antioxidants, 9 mL of phosphate buffer (pH 7.3) was added to 1 mL of a 1.00 mM antioxidant solution. Between measurements, the working electrode was polished with 0.05 µm alumina powder on a polishing cloth and rinsed with water.

### 3.6. Preparation of Large Unilamellar Vesicles (LUVs)

PC soy or DMPC liposomes were used as membrane mimetic systems. In order to evaluate the antioxidant activity of the AOs and their incorporation in the membrane, the AOs were incubated with preformed liposomes or incorporated during the liposome preparation (also known as phytosomes).

Liposomes were prepared using the hydration–extrusion method, as previously described [20]. PC soy or DMPC lipids were dissolved in chloroform in a round-bottom flask, and the solvent was evaporated. The lipidic films were then left under vacuum for 3 h. The lipidic films were hydrated by adding PBS (0.01 M, pH 7.4) to form large multilamellar vesicles (MLVs). MLVs were then subject to 5 cycles of freezing/thaw. The resulting suspensions were extruded through a 100 nm pore size polycarbonate filter (Whatman), using a LIPEX Biomembrane extruder at 37 ± 0.1 °C for 10 cycles to obtain LUVs with 100 nm of diameter. The final suspension was kept at 4 °C until use.

In order to evaluate the antioxidant activity with AOs incorporated in the liposome, an ethanolic solution (final concentration 0.75 μM) was added to the chloroform solution during the PC soy liposomes preparation.

For quenching assays, fluorescence probes were incorporated in the liposomes through the addition of each probe, with a probe–lipid ratio of 1:300 (mol/mol) for DPH or TMA-DPH and 1:100 (mol/mol) for 2-AS, in the initial DMPC chloroform solution.

For anisotropy assays, the incorporation of AOs and fluorescence probes (DPH or TMA-DPH) in the liposomes was performed by adding each probe, in a probe–lipid ratio of 1:300 (mol/mol), and each polyphenol as an ethanolic solution (final concentration, 200 μM), in the initial DMPC chloroform solution.

### 3.7. Dynamic Light Scattering (DLS) Measurements

Dynamic light scattering (DLS) was used to measure the average size and polydispersity index (P.I.) of liposome suspensions, with a Zetasizer Nano-ZS instrument (Malvern Instruments, version 7.12, Malvern, UK). Samples were back scattered by a helium-neon laser (633 nm) at an angle of 173°, refractive indices of 1.330 for PBS (water) and 1.400 for lipids, and viscosity of the dispersant of 0.6913 cP (water), at a constant temperature of 37 ± 0.1 °C. The inter-day variability in liposome preparations was lower than 5%, and the P.I. of all the prepared liposome suspensions was lower than 0.1.

### 3.8. Fluorescence Quenching Measurements

The insertion of the synthetized and parental compounds in the LUVs membrane was evaluated through a fluorescence quenching assay, using three fluorescence probes (DPH, TMA-DPH and 2-AS, located at 7.8, 10.9 and 16.8 Å, respectively, from the center of the membrane) [19,20,36]. Cholesterol and α-Tocopherol were used as reference compounds. The assays were performed on a Varian Cary Eclipse spectrofluorometer using a 1 cm quartz cuvette. Excitation and emission wavelengths of 360/427 nm, 365/426 nm and 363/458 for DPH, TMA-DPH and 2-AS, respectively, were used, with a slit width of 5 nm for excitation and 5 nm for emission, at the speed of 120 nm/min with data intervals of 1 nm. The DMPC liposome suspension at 1 mM, containing each probe, was kept at 37 ± 0.1 °C for thermal equilibrium. Then, the fluorescence quenching studies were started at time 0 min and after 4 min, 20 μL of AO solution (final concentration of 200 μM) was added. The fluorescence was measured over further 26 min. Three independent measurements were obtained for each compound and each tested probe.

### 3.9. Effect of Compounds on the Fluorescence Polarization of Probes

Two fluorescence probes (DPH and TMA-DPH) were used in the fluorescence anisotropy determinations [20,36]. Each probe was mixed with DMPC before MLV preparation in a molar ratio of 300 lipid–probe, and the synthetized compounds, cholesterol or α-tocopheroll, were incorporated in the liposomes at 200 μM, as previously described. Fluorescence anisotropy determinations were also performed with a Varian Cary Eclipse spectrofluorometer using a 1 cm quartz cuvette. Excitation and emission wavelengths of 360/427 nm and 365/426 nm for DPH and TMA-DPH, respectively, were used, with a slit width of 5 nm for excitation and 5 nm for emission. The steady-state anisotropy <r> values were calculated according to [37]:

$$<r>=\frac{I_{VV}-I_{VH}G}{I_{VV}+2\;I_{VH}G}$$

where $I_{VV}$ and $I_{VH}$ represent the emission intensities when the emission polarizer was oriented vertical (parallel) and horizontal (perpendicular) to the polarization of the excitation light. G is a correction factor and is given by the ratio of vertical to horizontal components when the excitation light is polarized in the horizontal direction, G= $I_{HV}/I_{HH}$. The anisotropy values are then given as the mean of the values of two independents assays.

### 3.10. Evaluation of the Antioxidant Activity of Compounds in PC Liposomes

The antioxidant activity of cholesteryl esters and their parental phenolic acids was evaluated in PC soy liposomes LUVs prepared without the incorporation and with the incorporation (final concentration 0.75 μM) of these compounds. Here, 250 μL of PC soy LUVs (final concentration of 175 μM) was added to a UV-Star^®^ 96-well microplate (Greiner Bio-one, Merck, Lisbon). In the case of PC soy liposomes that were not prepared with the antioxidant compounds, LUVs were incubated at 37 °C for 10 min, and a phenolic DMSO solution (final concentration of 0.75 μM) was added and incubated for a further 10 min. Lipid peroxidation was then initiated by the addition of AAPH in PBS (final concentration of 0.40 μM). The increase in conjugated diene concentration was determined by measuring the absorbance of each sample at 234 nm every 5 min over 24 h at 37 °C using a Powerwave XS Microplate Reader (Bio-Tek Instruments, Soquimica, Lisbon, Portugal) [19,20]. The well-known natural biomembrane antioxidant α-tocopherol was also tested. The results are shown as the time (min) needed for an increase of 0,4% in the conjugated diene concentration of each sample. Each compound was tested in triplicate in four independent experiments.

### 3.11. Statistical Analysis

Statistical analysis was performed using SPSS 29.0 software. One-way analysis of variance (ANOVA) with Dunkan’s test was used, with the level of significance set at *p* < 0.05.

## 4. Conclusions

A one-step esterification procedure for the preparation of a series of novel phenolipids was developed, yielding cholesteryl esters of polyphenolic acids in moderate to high yields (50–70%). Alternatively, cholesteryl caffeate was also obtained in high yield (>95%) with a Verley–Doebner modification of the Knoevenagel condensation. Preliminary characterization of the new compounds in terms of solubility and radical scavenging capacity showed that these compounds have a similar liposolubility and a better or comparable radical scavenging capacity to that of the natural membrane antioxidant α-tocopherol. Cyclic voltammetry confirmed lower anodic peak potentials for the cholesteryl esters when compared with the corresponding non-esterified phenolic acids. Despite having strong antiradical capacity and being able to penetrate membranes, the antioxidant efficiency of cholesteryl esters in liposomal systems was deeply dependent on the phenolipid polyphenolic moiety structure. In the phosphatidylcholine LUVs system tested, cholesteryl phenolipids with a more rigid structure, PCA-Chol and CA-Chol, when incorporated in the liposomes showed remarkable antioxidant capacity when compared with α-tocopherol, while DHCA-Chol and DOPAC-Chol did not show any improvement in the oxidative stability of LUVs when compared with α-tocopherol. However, in ex vivo and in vivo studies, different results may be obtained and, as all the cholesteryl esters showed at least a similar activity when compared with α-tocopherol, all compounds can still be considered strong candidates for future interventions against oxidative stress in cells and lipoproteins. Evaluation of their antioxidant and anti-inflammatory capacity is now being performed. Caffeic acid also showed a remarkable antioxidant capacity in the liposomal system used. However, this compound did not show an important protective activity against oxidative injury in cells [20]. Therefore, this phenolic acid is a good candidate to for use as an antioxidant ingredient in dermal and cosmetic liposomal preparations.

In conclusion, cholesteryl phenolipids are promising candidates for the protection of biomembranes to a greater extent than α-tocopherol.

## Figures and Tables

**Table 1:** EC_50_ (mol compound/mol DPPH^●^), and miLog *p* values.

Compound	miLog *P*	EC_50_ *		Compound	miLog *P*	EC_50_ *	
		5 min	30 min			5 min	30 min
Cholesterol	7.9	-	-	α-Tocopherol	9.0	0.33	0.29
PCA	0.86	0.22	0.19	PCA-Chol	8.8	0.24	0.14
DOPAC	0.39	0.13	0.13	DOPC-Chol	8.8	0.26	0.23
DHCA	0.91	0.19	0.13	DHCA-Chol	8.9	0.26	0.26
CA	0.94	0.23	0.21	CA-Chol	9.0	0.30	0.29

* Standard deviations were lower than 5%.

**Table 2:** Anodic peak potential (*Epa*), cathodic peak potential (*Epc*), anodic peak current (*Ipa*) and cathodic peak current (*Ipc*) of phenolic acids and their cholesteryl esters.

Compound	*Epa* (V)*	*Epc* (V) *	*Ipa* (µA) *	*Ipc* (µA) *	Compound	*Epa* (V) *	*Ipa* (µA) *
CA	0.289	0.030	23.43	12.74	CA-Chol	0.236	4.16
DHCA	0.422	−0.073	17.36	10.67	DHCA-Chol	0.191	3.35
DOPAC	0.364	−0.027	20.26	13.64	DOPAC-Chol	0.170	4.75
PCA	0.525	−0.007	18.60	8.14	PCA-Chol	0.289	4.07

* Standard deviations were lower than 5%.

## Data Availability

Data are contained within the article and Supplementary Materials.

## Supplementary Materials

The following supporting information can be downloaded at https://www.mdpi.com/article/10.3390/molecules29204959/s1, Proton and Carbon Nuclear Magnetic Resonance spectra of cholesteryl caffeate, dihydrocaffeate, homoprotocatechuate and protocatechuate.
