# Supplementary material for: Cholesteryl Phenolipids as Potential Biomembrane Antioxidants
Source: Molecules. 2024 Oct 20;29(20):4959. doi: 10.3390/molecules29204959 (PMC11510111; doi:10.3390/molecules29204959)
Supplement: Supplementary file 1 [file molecules-29-04959-s001.zip › molecules-3263488-supplementary.pdf]

Supplementary Materials

Cholesteryl Phenolipids as Potential Biomembrane Antioxidants

Vânia Costa [1], Marlene Costa [1], Francisca Arques [1], Mariana Ferreira [1], Paula Gameiro [1], Dulce Geraldo [2], Luís S. Monteiro [2], Fátima Paiva-Martins [1,*]

[1] REQUIMTE-LAQV, Department of Chemistry and Biochemistry, Faculty of Sciences, University of Porto, Portugal, mpmartin@fc.up.pt

[2] Chemistry Centre, University of Minho, Gualtar, 4710-057 Braga, Portugal; monteiro@quimica.uminho.pt

* Correspondence: mpmartin@fc.up.pt

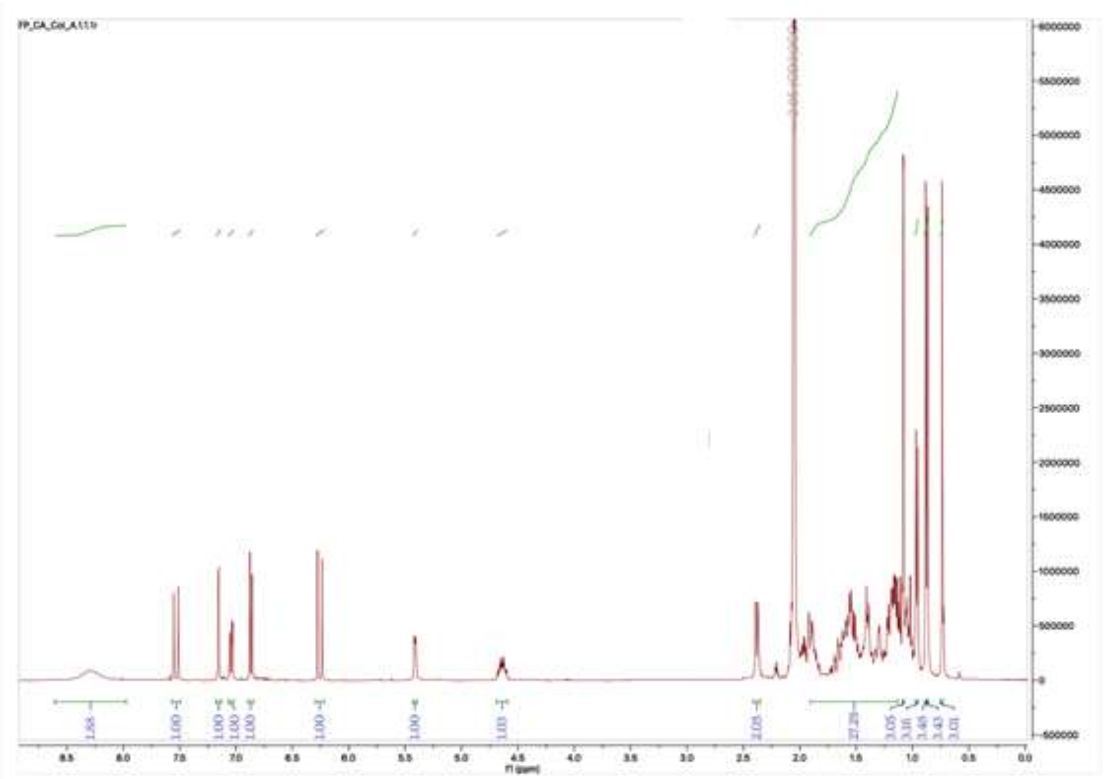

Figure S1 – ^{1}H-NMR spectra (400 MHz, $(CD_3)_2CO$) of cholesteryl caffeate.

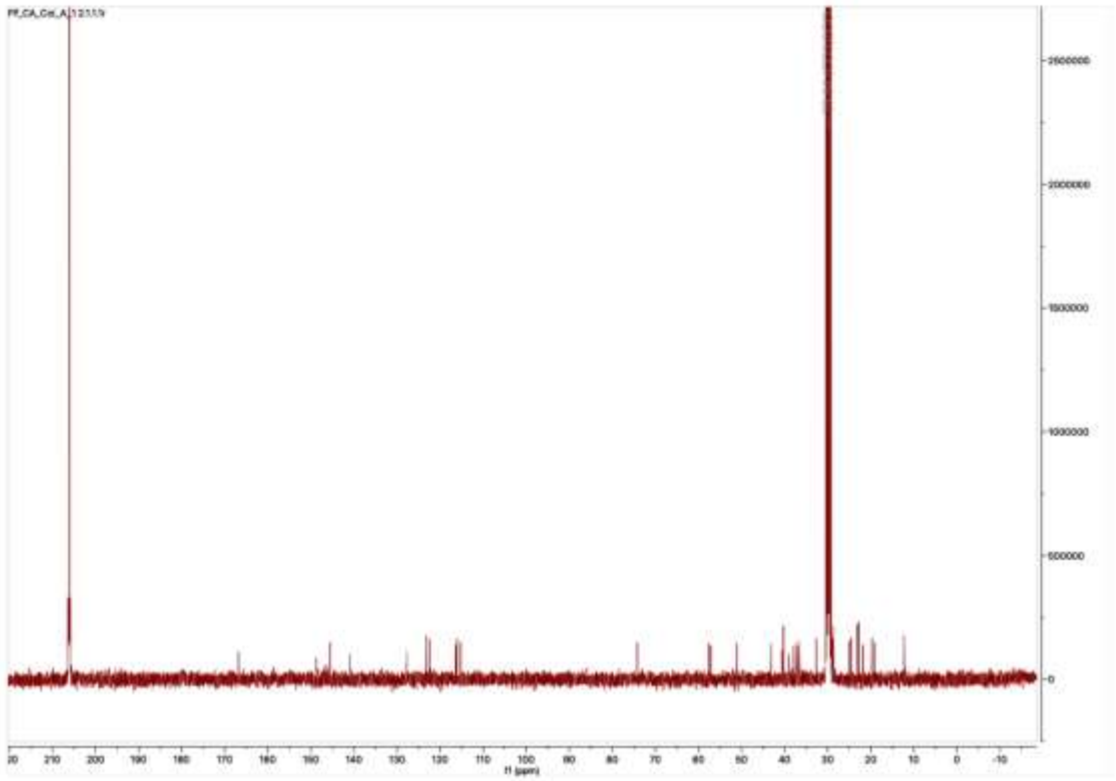

Figure S2 – ^{13}C-NMR spectra (100 MHz, $(CD_3)_2CO$) of cholesteryl caffeate.

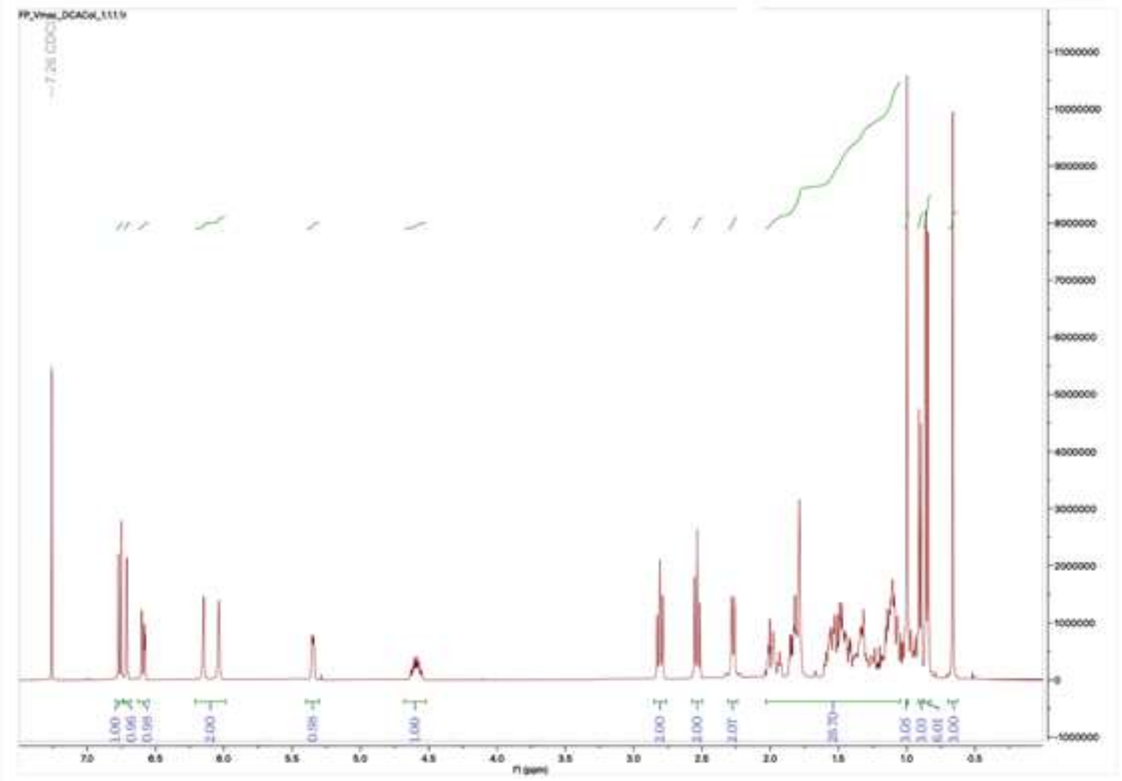

Figure S3 – ^{1}H-NMR spectra (400 MHz, $CDCl_3$) of cholesteryl dihydrocaffeate.

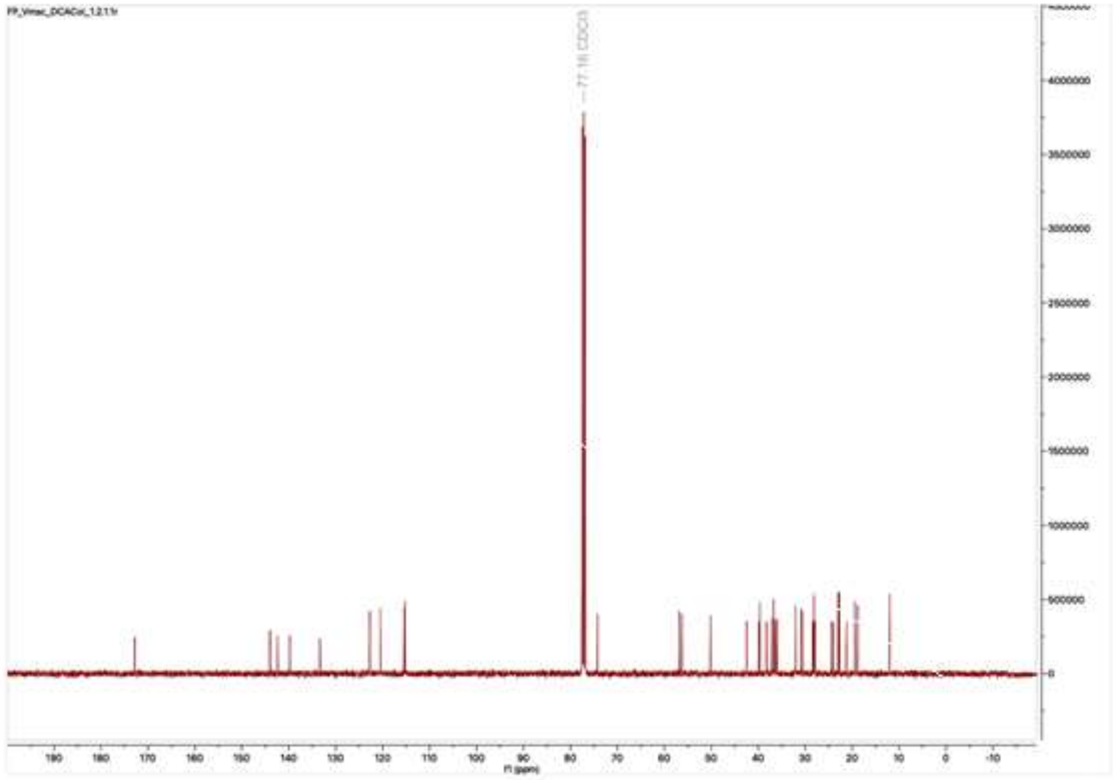

Figure S4 – ^{13}C-NMR spectra (100 MHz, $CDCl_3$) of cholesteryl dihydrocaffeate.

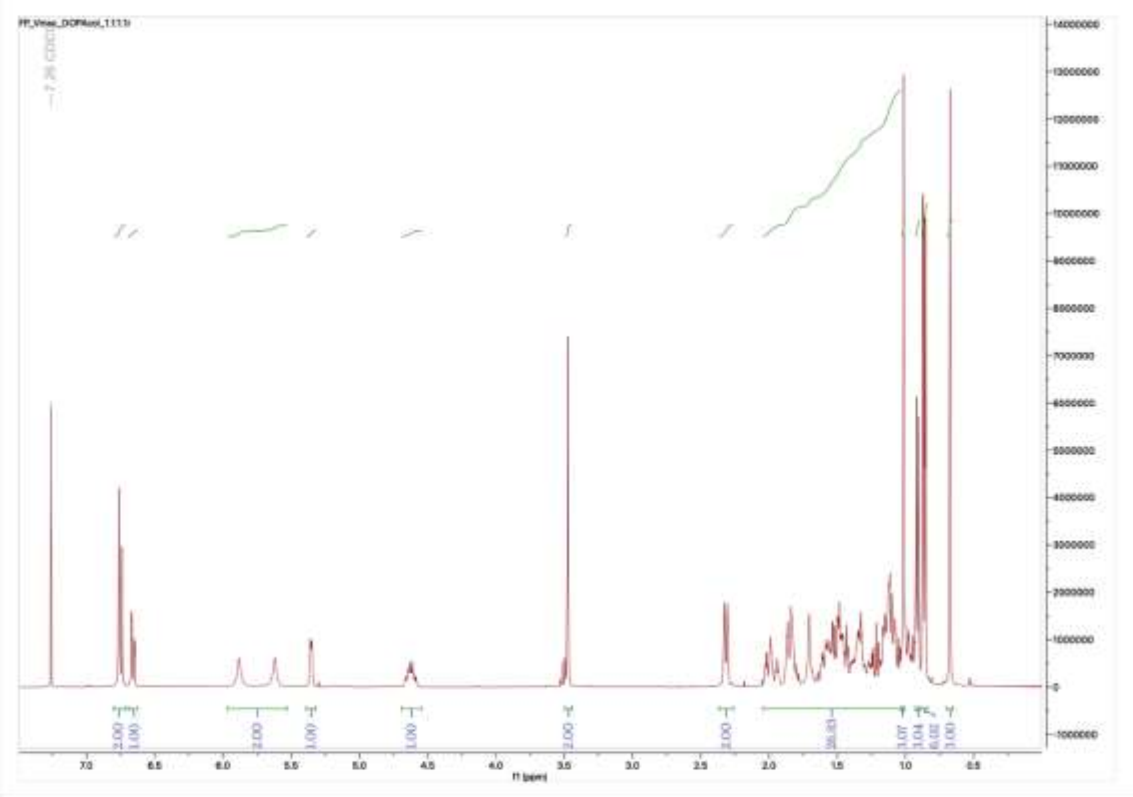

Figure S5 – ^{1}H-NMR spectra (400 MHz, $CDCl_3$) of cholesteryl homoprotocatechuate.

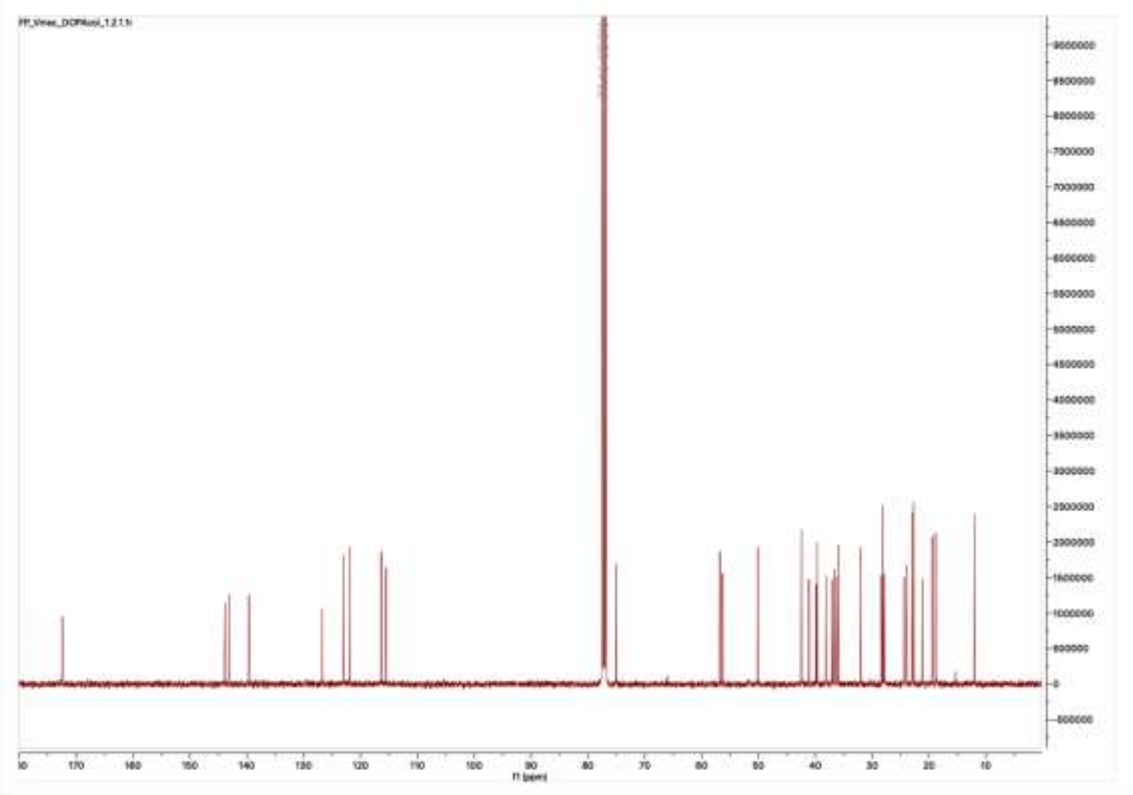

Figure S6 – ^{13}C-NMR spectra (100 MHz, $CDCl_3$) of cholesteryl homoprotocatechuate.

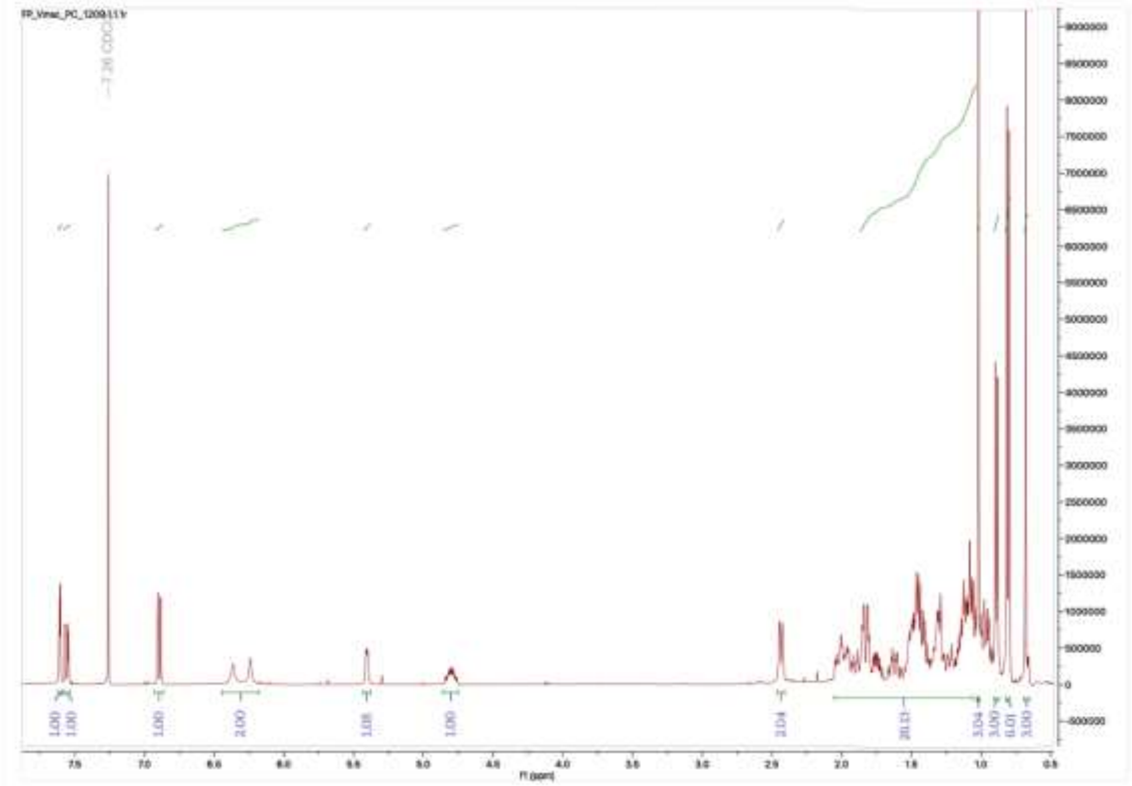

Figure S7 – ^{1}H-NMR spectra (400 MHz, $CDCl_3$) of cholesteryl protocatechuate.

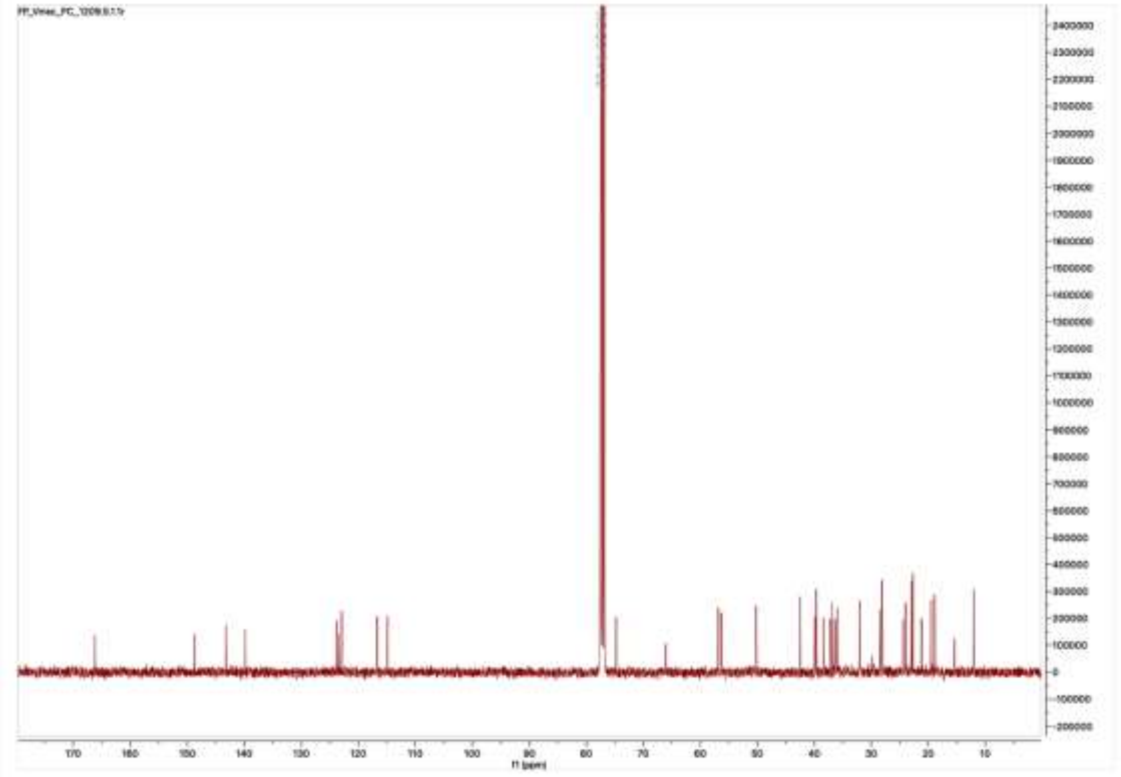

Figure S8 – ^{13}C-NMR spectra (100 MHz, $CDCl_3$) of cholesteryl protocatechuate.
